# Blue-Light Exposure Enhances Productivity, Total Phenolic Content, and Sensory Attributes in Edible Mushrooms: A Multispecies Study

**DOI:** 10.1007/s11130-026-01555-4

**Published:** 2026-08-06

**Authors:** Atahualpa Guzmán-de-Casa, Eréndira Valencia-Avilés, Pedro A. García-Saucedo, Erick Sierra-Campos, Teresita del Carmen Ávila-Val, Víctor Gómez-Reyes, Víctor Olalde-Portugal

**Affiliations:** 1 Depto. de Biotecnología y Bioquímica, Centro de Investigación y de Estudios Avanzados del IPN, Irapuato, Gto 36824 México; 2 Universidad Politécnica de Uruapan, Uruapan, Michoacán 60210 México; 3 Facultad de Agrobiología ‘Presidente Juárez’, Universidad Michoacana de San Nicolás de Hidalgo, Uruapan, Michoacán 60040 México; 4 Facultad de Ciencias Químicas, Universidad Juárez de Durango, Gómez Palacio, Durango 35010 Mexico; 5 Facultad de Biología, Universidad Michoacana de San Nicolás de Hidalgo, Morelia, Michoacán 58030 Mexico

**Keywords:** Check-All-That-Apply (CATA) analysis, Antioxidant activity, Biological efficiency

## Abstract

Cultivated mushrooms are increasingly recognized as sustainable food resources and strategic components of global food security and circular economy systems because of their high nutritional and nutraceutical value, desirable sensory characteristics, and capacity to transform lignocellulosic residues into high-value food products. This study evaluated the effects of blue-light exposure during cultivation on productivity, morphology, total phenolic content (TPC), antioxidant activity, and sensory attributes of two wild edible mushrooms (*Hericium erinaceus* and *Neolentinus ponderosus*) and three commercial species (*Lentinula edodes*, *Pleurotus djamor*, and *P. ostreatus*) cultivated on oak sawdust-based substrate. Sensory characterization was performed using the Check-All-That-Apply (CATA) methodology with a semi-trained panel. Blue light significantly increased biological efficiency in a species-dependent manner, with the strongest responses observed in *P. djamor* (428%), *P. ostreatus* (122%), and *H. erinaceus* (43%). Blue-light exposure promoted normal fruiting body development and pigmentation, while cultivation in darkness resulted in elongated stipes, reduced pigmentation, and poorly differentiated basidiocarps, particularly in *P. djamor*. CATA and hedonic analyses successfully discriminated sensory differences among species and cultivation conditions. Blue-light exposure increased TPC in *N. ponderosus* and reduced it in *P. ostreatus*, whereas antioxidant activity showed only minor variations among incubation conditions. Collectively, the results demonstrate that incubation under blue light, relative to darkness, modulates morphological traits, TPC content, and sensory attributes, highlighting its potential as a practical tool for optimizing the production for targeted market segments.

**Supplementary Information:**

The online version contains supplementary material available at https://doi.org/10.1007/s11130-026-01555-4.

## Introduction

Mushroom cultivation is increasingly recognized as a sustainable strategy to enhance food and nutritional security. This is particularly relevant in developing countries such as Mexico, where the high prevalence of cardiovascular diseases, diabetes, hypertension, and malnutrition is associated with inadequate dietary patterns [1]. Edible mushrooms are valued not only for their nutritional quality but also for their bioactive compounds, including phenolics, polysaccharides, vitamins, antioxidants, and mycochemicals, which contribute to human health and disease prevention. In addition, mushroom production supports several Sustainable Development Goals (SDGs) by promoting circular bioeconomy practices through the bioconversion of agricultural and forestry residues into high-value food and nutraceutical products for humans and animals while generating employment and reducing environmental impacts [2].

The global mushroom industry has expanded rapidly, reaching an estimated market value of USD 55 billion and production volumes of approximately 48 and 49 million tons in 2022 and 2024, respectively [3]. Despite the existence of more than 2,200 edible macrofungal species, only about 100 are cultivated commercially, indicating considerable untapped potential for the diversification and conservation of fungal genetic resources [4]. Commercial production is dominated by genera such as *Lentinula*, *Pleurotus*, *Auricularia*, *Agaricus*, and *Flammulina* [5]. In Mexico, cultivation focuses mainly on *Agaricus* and *Pleurotus*, whereas native species with recognized culinary and functional value remain largely underexplored despite the country’s rich fungal biodiversity and ethnomycological heritage [6, 7]. Among these, *Hericium erinaceus* and *Neolentinus* spp. are particularly noteworthy for their sensory attributes, cultural significance, and nutraceutical value.

The productivity and quality of cultivated mushrooms are strongly influenced by substrate composition and environmental conditions. Factors such as carbon-to-nitrogen ratio, mineral availability, moisture content, temperature, humidity, gaseous composition, and light can affect mycelial growth, fruiting body development, and the accumulation of bioactive compounds [8, 9]. Among these factors, light plays a central role in regulating fungal morphogenesis and metabolism. Blue-light photoreceptors control developmental processes associated with primordia formation, fruiting body differentiation, and the activation of metabolic pathways involved in carbohydrate utilization, lignocellulose degradation, and secondary metabolite biosynthesis [10–13]. Inadequate light conditions may therefore impair normal development and alter mushroom quality.

Although the role of light in fruiting body induction is well established, increasing attention has recently been directed toward its potential to enhance mycelial growth in submerged cultivation and to modulate its nutraceutical properties, including lipid composition, polysaccharide production, and the aromatic profile [14]. Previous studies have reported that specific light spectra can modify gene expression, acting as a metabolic regulator and influencing morphological differentiation, biomass production, biological efficiency, enzymatic activity, biosynthesis of mycochemicals, phenolic accumulation, antioxidant activity, and other quality-related traits in a mushroom species-dependent manner, with blue light often yielding the most favorable responses [15–17]. However, comparative studies across multiple edible mushroom species remain limited, particularly those integrating production performance, nutraceutical characteristics, and sensory quality.

Cultivation practices, including substrate composition and environmental conditions, may influence sensory attributes such as appearance, flavor, texture, and overall acceptability [18]. While descriptive sensory analysis provides detailed product characterization, it requires extensive panel training. In contrast, the Check-All-That-Apply (CATA) methodology offers a rapid and reliable alternative for identifying sensory differences using semi-trained or untrained panelists and has been successfully applied to mushroom-based products [19].

Therefore, the objective of this study was to evaluate the effects of blue-light exposure during cultivation, compared with darkness, on productivity, morphological traits, total phenolic content, and antioxidant activity in five edible mushroom species, including both wild and commercial strains. In addition, sensory differences associated with species and cultivation conditions were assessed using the CATA methodology and a semi-trained sensory panel.

## Materials and Methods

### Mushroom Cultivation and Sample Preparation

Dikaryotic strains of *N. ponderosus* and *H. erinaceus* were isolated from wild fruiting bodies collected in Michoacán, Mexico, whereas commercial strains of *P. ostreatus*, *P. djamor*, and *L. edodes* were obtained from local producers. All specimens were taxonomically identified and deposited in the Herbarium of the Universidad Michoacana de San Nicolás de Hidalgo (*P. djamor* EBUM-F 01417, *L. edodes* EBUM-F 01418, *P. ostreatus* EBUM-F 01415, *H. erinaceus* EBUM-F 01412, and *N. ponderosus* EBUM-F 01411).

*Hericium erinaceus*,* P. ostreatus*,* P. djamor*, and *L. edodes* were cultivated (*n* = 5) on oak sawdust supplemented with 15% wheat bran [20, 21], whereas *N. ponderosus* was grown on a 1:1 (w/w) mixture of oak and pine sawdust (Supplementary 1.1). Substrates were adjusted to 60% moisture, packed into 1.5-kg portions, sterilized at 121 °C for 90 min, and inoculated with sorghum grain spawn. Cultures were incubated either in darkness or under blue light (25 µmol m⁻² s⁻¹, 8 h photoperiod). Fruiting bodies were harvested and dehydrated at 45 °C for 16 h. At the end of the harvest period, the accumulated data were used to calculate total yield and the biological efficiency (BE). BE is the ratio of fresh fruiting body weight (g) *per* dry weight of substrates (g), expressed as a percentage.

### Extraction and Chemical Analyses

Dried mushrooms were ground into a fine powder (approximately 180 μm particle size) and sequentially extracted with hot water (100 °C, 2 h) and 80% ethanol (25 °C, 24 h). Total phenolic content (TPC) was determined using the Folin–Ciocalteu assay and expressed as mg gallic acid equivalents *per* g dry weight (mg GAE/g DW). Antioxidant activity was evaluated using 2, *2-diphenyl-1-picrylhydrazyl *(DPPH•) and 2’-azino-bis(3-ethylbenzothiazoline-6-sulfonic acid (ABTS•⁺) radical scavenging assays and expressed as percentage inhibition [15].

### Sensory Evaluation

Sensory characterization was performed using a semi-trained panel consisting of individuals who had received basic to intermediate training in evaluating certain sensory attributes (Supplementary 1.2). Panelists were selected for their sensitivity to the bitterness of 6-n-propylthiouracil (PROP), and then trained in taste recognition and sensory discrimination in accordance with ISO 3972 [22] and ISO 5496 [23] guidelines. Before evaluation, dehydrated mushroom samples were rehydrated, coded, and presented in a randomized order. Samples were assessed using the Check-All-That-Apply (CATA) methodology, considering attributes related to appearance (brown color), flavor (cereal-like and mushroom flavors), basic tastes (sweet, salty, bitter, and sour), texture (hardness, fibrousness, elasticity, and chewiness), and mouthfeel (astringent and pungent sensations) [18]. Overall acceptability was evaluated using a 9-point hedonic scale. The final sensory evaluation was conducted by 27 semi-trained panelists (17 women and 10 men), aged between 17 and 44 years.

### Statistical Analysis

Differences in biomass production, total phenolic content, and antioxidant activity between the two cultivation conditions (blue light and darkness) were evaluated within each species using one-way ANOVA followed by Tukey’s *post hoc* test (*n* = 5; *p* < 0.05). CATA data were analyzed using Cochran’s Q test followed by Sheskin’s critical difference procedure. Associations between samples and sensory descriptors were evaluated using chi-square tests of independence and principal coordinates analysis (PCoA). All sensory statistical analyses were performed using XLSTAT.

## Results and Discussion

### Effects of Blue Light on Fruiting Body Morphology and Productivity

**Fig. 1:**
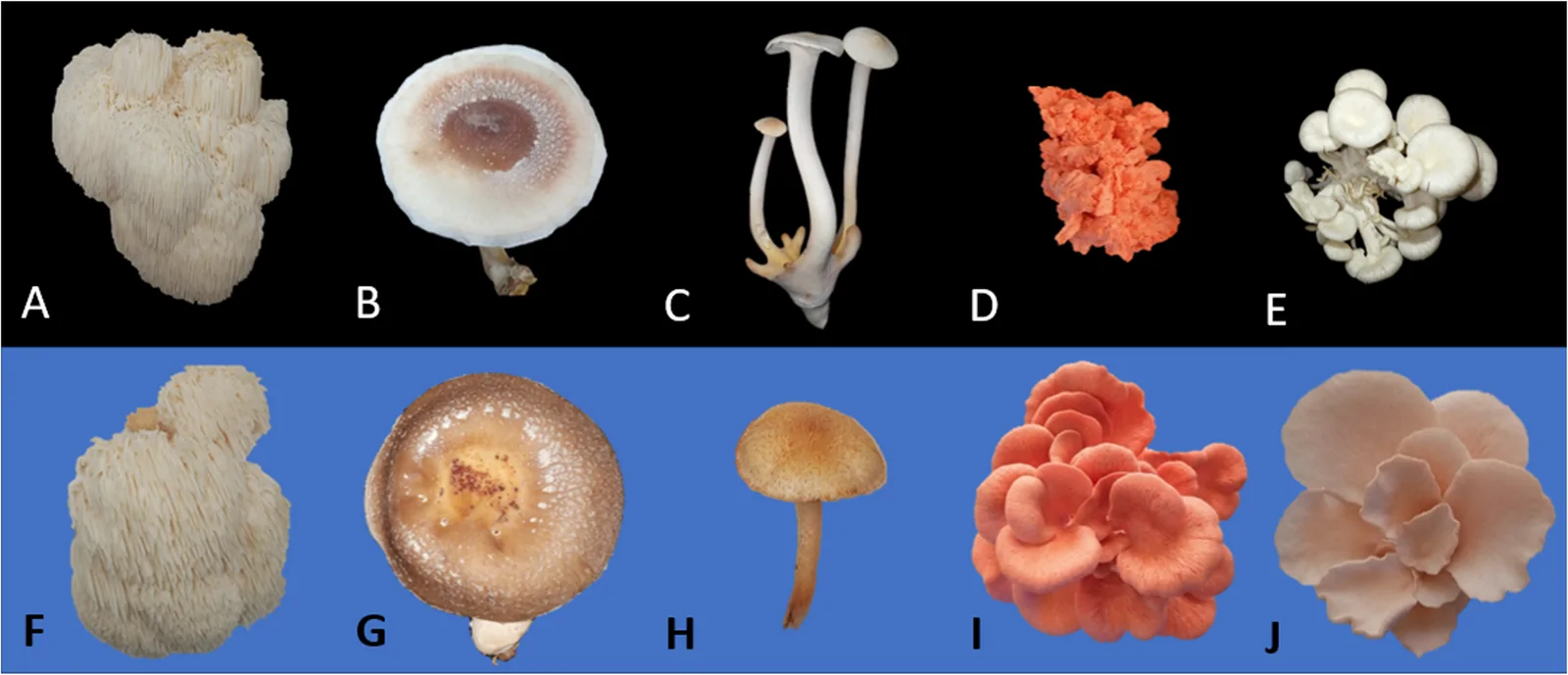
Fruiting body morphology of edible mushrooms *H. erinaceus *(**A**, **F**), *L. edodes* (**B**, **G**), *N. ponderosus* (**C**, **H**), *P. djamor* (**D**, **I**), and *P. ostreatus* (**E**, **J**) cultivated under blue-light exposure or in complete darkness. Darkness (**A**-**E** panels) and blue light exposure (**F**-**J** panels)

Blue-light exposure markedly influenced fruiting body development in most species (Fig. 1). *Pleurotus ostreatus*, *P. djamor*, *L. edodes*, and *N. ponderosus* produced well-differentiated basidiocarps with typical morphology and pigmentation under blue light. In contrast, cultivation under dark conditions resulted in elongated stipes, reduced pigmentation, and, in some cases, malformed fruiting bodies, particularly in *P. djamor* (Fig. 1d). The limited morphological response of *H. erinaceus* contrasts with the pronounced effects of blue light on pileus development and pigmentation observed in most agaricoid mushrooms. This difference may be related to its distinctive spine-forming morphology, which reduces the visible impact of light-mediated morphogenesis. Nevertheless, the larger fruiting bodies and higher biomass production under blue light (Table 1) suggest that light still promotes physiological processes associated with fruiting body development, as previously reported for *H. coralloides* [24].

**Table 1:** Biomass productivity and biological efficiency (BE) of wild and commercial mushrooms cultivated under blue-light exposure and in darkness^a, b^

Mushroom	Productivity (g/bag)		Biological Efficiency		Increase (%)
	Darkness	Blue light	Darkness	Blue light	
*P. ostreatus*	176.2 ± 49.5^b^	391.8 ± 56.9^a^	35.2 ± 9.9^b^	78.2 ± 11.4^a^	122%
*P. djamor*	58.8 ± 21.0^d^	310.8 ± 38.0^ab^	11.6 ± 4.2^b^	62.2 ± 7.6^ab^	428%
*L. edodes*	311.7 ± 43.5^ab^	349.2 ± 38.0^a^	62.3 ± 8.7^ab^	69.8 ± 7.5^a^	12%
*H. erinaceus*	134.8 ± 29.3^c^	192.7 ± 25.7^b^	27.0 ± 5.9^c^	38.5 ± 5.1^b^	43%
*N. ponderosus*	111.6 ± 24.7^cd^	125.8 ± 22.9^cd^	22.3 ± 4.9^cd^	25.2 ± 4.6^cd^	13%

^a^Values represent mean ± standard deviation (SD). ^b^Different letters within each species indicate significant differences according to ANOVA followed by Tukey *post hoc* tests (*n* = 5, *p* < 0.05)

These findings confirm the essential role of light in fruiting body differentiation and are consistent with previous studies describing blue light as a major regulator of basidiomycete morphogenesis [8, 24, 25]. The morphological abnormalities observed under dark conditions suggest impaired photomorphogenic development, emphasizing the essential role of appropriate light management in promoting optimal productivity and the development of commercially desirable fruiting body characteristics [26, 27].

Blue light also enhanced productivity, although responses varied among species (Table 1). The greatest increase in biological efficiency (BE) was observed in *P. djamor* (428%), followed by *P. ostreatus* (122%) and *H. erinaceus* (43%), whereas *L. edodes* and *N. ponderosus* exhibited only modest improvements (12–13%). While agaricoid species such as *Pleurotus* spp. exhibited pronounced morphological and productivity responses to blue-light exposure, *H. erinaceus* showed only limited morphological changes despite an increase in biomass production. This finding suggests that physiological responses to light can occur even in the absence of noticeable morphological modifications. The results indicate that blue light functions not only as a developmental signal but also as a regulator of biomass allocation toward fruiting body formation. Similar responses have been reported in other cultivated mushrooms, where blue light promotes reproductive development and enhances yield, often leading to the production of fewer but larger fruiting bodies, as observed in *P. ostreatus* (Fig. 1). The species-specific differences observed in morphology and productivity may be attributed to variations in photoreceptor-mediated signaling pathways, fruiting body architecture, and metabolic regulation mechanisms [24, 26, 28].

Although the underlying mechanisms were not investigated in the present study, previous research suggests that these responses may be associated with enhanced energy metabolism and carbon flux through the activation of genes involved in ATP biosynthesis and primary metabolism [11, 29, 30]. The marked differences among species highlight the importance of species-specific cultivation protocols when implementing light-based production strategies.

### Total Phenolic Content and Antioxidant Activity

Total phenolic content (TPC) ranged from 1.6 to 4.1 mg GAE g⁻¹ DW and exhibited species-specific responses to blue-light exposure (Table 2). TPC increased significantly in *N. ponderosus* and decreased in *P. ostreatus*, whereas no significant effects were detected in *H. erinaceus*, *P. djamor*, or *L. edodes*. These findings suggest that the regulation of phenolic compound accumulation under blue light varies among mushroom species.

**Table 2:** Total phenolic content and antioxidant activity of mushroom fruiting bodies cultivated under blue light and dark conditions^a, b,c^

Mushroom	TPC (mg GAE/g dw)		DPPH. (inhibition %)		ABTS^+^ (Inhibition %)	
	Darkness	Blue light	Darkness	Blue light	Darkness	Blue light
*P. ostreatus*	3.3 ± 0.3^ab^	2.4 ± 0.3^cd^	45.3 ± 7.1^bc^	40.5 ± 7.8^cd^	50.9 ± 7.0^bc^	53.5 ± 6.0^bc^
*P. djamor*	3.8 ± 0.4^a^	4.1 ± 0.3^a^	44.9 ± 4.5^bc^	46.4 ± 2.6^bc^	48.4 ± 4.3^c^	53.4 ± 3.0^bc^
*L. edodes*	2.6 ± 0.4^cd^	2.3 ± 0.3^de^	28.9 ± 3.7^e^	33.5 ± 3.5^de^	60.7 ± 6.5^ab^	64.7 ± 3.1^a^
*H. erinaceus*	1.6 ± 0.3^f^	1.9 ± 0.3^ef^	56.0 ± 3.5^ab^	62.0 ± 6.6^a^	45.0 ± 3.5^c^	41.0 ± 3.8^c^
*N. ponderosus*	2.0 ± 0.3^ef^	3.0 ± 0.2^bc^	43.9 ± 7.1^bc^	40.2 ± 3.7^cd^	51.1 ± 5.0^bc^	49.7 ± 4.1^c^

^a^Total phenolics (TPC) are expressed as mg of gallic acid equivalents per gram of dry weight. Antioxidant activity is expressed as percentage inhibition in the DPPH and ABTS•+ radical scavenging assays. ^b^Data are expressed as mean ± standard deviation (SD). ^c^Means followed by different letters within each species are significantly different according to one-way ANOVA followed by Tukey *post hoc* tests (*n* = 5, *p* < 0.05)

Enhanced phenolic accumulation in *N. ponderosus* under blue light may represent activation of shikimate-derived routes as an adaptive response to light-induced oxidative stress, as previously reported for some mushrooms [15]. Conversely, reductions in TPC in *P. ostreatus* may reflect differences in carbon partitioning toward sporome growth or pigmentation processes [29, 30]. Importantly, despite changes in phenolic content, antioxidant activity measured by DPPH. and ABTS.^**+**^ assays remained relatively stable across treatments. This suggests that antioxidant capacity depends on multiple bioactive compounds, including phenolics, β-glucans, ergothioneine, and other antioxidant metabolites commonly found in edible mushrooms [31]. These results indicate that blue-light exposure can improve TPC content, although the magnitude and direction of the response depend on the mushroom species.

### Sensory Characterization and Consumer Acceptance

**Fig. 2:**
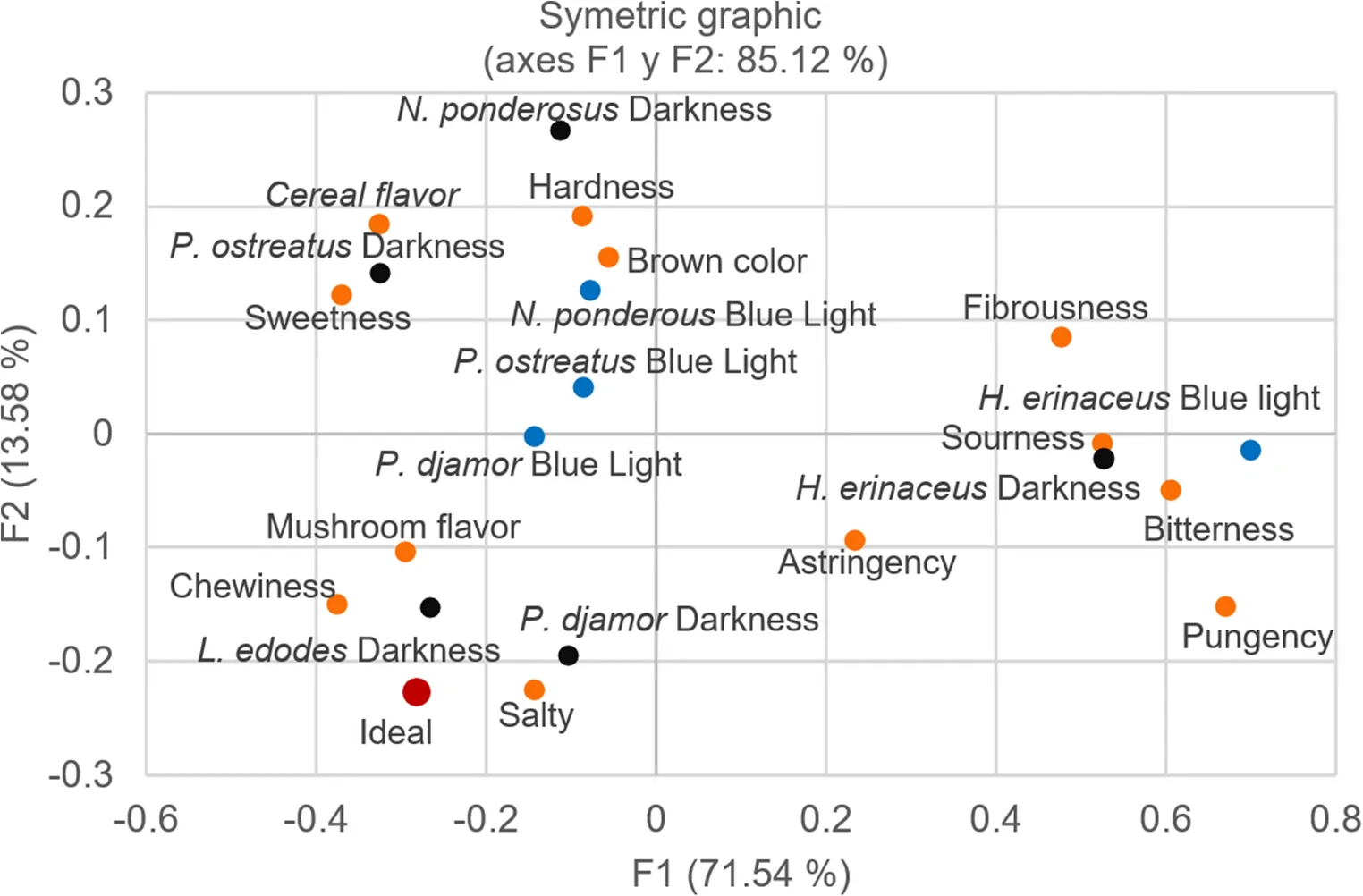
Symmetrical Principal Component Analysis (PCA) biplot of sensory attributes and mushroom samples

The CATA methodology effectively discriminated sensory attributes among mushroom species (*p* < 0.05), particularly bitterness, sourness, mushroom flavor, and texture-related descriptors (Table 3; Fig. 2). These findings confirm that CATA is a rapid, reliable, and practical method for sensory characterization of edible mushrooms without the need for highly trained panelists [18].

**Table 3:** Pairwise comparisons among mushroom samples for CATA attributes using Sheskin’s critical difference procedure^a^

Attributes	*P*. ostreatus		*N*. ponderosus		H. erinaceus		*P*. djamor		L. edodes
	Darkness	Blue light	Darkness	Blue light	Darkness	Blue light	Darkness	Blue light	Darkness
Sweetness	0.222 ^a^	0.111 ^a^	0.259 ^a^	0.296 ^a^	0.074 ^a^	0.037 ^a^	0.185 ^a^	0.259 ^a^	0.185 ^a^
Salty	0.444 ^ab^	0.630 ^ab^	0.296 ^a^	0.481 ^ab^	0.519 ^ab^	0.370 ^ab^	0.667 ^b^	0.630 ^ab^	0.667 ^b^
Bitterness	0.074 ^a^	0.296 ^a^	0.370 ^a^	0.296 ^a^	0.852 ^b^	0.926 ^b^	0.296 ^a^	0.259 ^a^	0.370 ^a^
Sourness	0.074 ^a^	0.148 ^ab^	0.111 ^a^	0.185 ^ab^	0.370 ^ab^	0.444 ^b^	0.111 ^a^	0.296 ^ab^	0.111 ^a^
Cereal-like flavor	0.630 ^b^	0.556 ^b^	0.593 ^b^	0.481 ^b^	0.259 ^ab^	0.074 ^a^	0.370 ^ab^	0.370 ^ab^	0.444 ^ab^
Mushroom	0.667 ^bc^	0.481 ^abc^	0.556 ^abc^	0.481 ^abc^	0.333 ^ab^	0.259 ^a^	0.704 ^c^	0.630 ^bc^	0.630 ^bc^
Brown color	0.963 ^c^	0.963 ^c^	0.963 ^c^	0.926 ^bc^	0.889 ^bc^	0.667 ^abc^	0.630 ^ab^	0.741 ^bc^	0.407 ^a^
Astringency	0.333 ^a^	0.370 ^a^	0.370 ^a^	0.481 ^ab^	0.778 ^b^	0.704 ^ab^	0.333 ^a^	0.333 ^a^	0.481 ^ab^
Pungency	0 ^a^	0.074 ^a^	0.037 ^a^	0.074 ^a^	0.148 ^a^	0.185 ^a^	0 ^a^	0.037 ^a^	0.074 ^a^
Hardness	0.370 ^a^	0.333 ^a^	0.593 ^a^	0.444 ^a^	0.222 ^a^	0.407 ^a^	0.333 ^a^	0.593 ^a^	0.296 ^a^
Fibrousness	0.370 ^a^	0.407 ^ab^	0.333 ^a^	0.296 ^a^	0.815 ^b^	0.815 ^b^	0.148 ^a^	0.333 ^a^	0.333 ^a^
Elasticity	0.519 ^a^	0.407 ^a^	0.519 ^a^	0.481 ^a^	0.333 ^a^	0.296 ^a^	0.630 ^a^	0.407 ^a^	0.519 ^a^
Chewiness	0.593 ^b^	0.519 ^ab^	0.370 ^ab^	0.370 ^ab^	0.185 ^a^	0.148 ^a^	0.593 ^b^	0.630 ^b^	0.593 ^b^

^a^Significant differences (*p* < 0.05) are indicated

**Fig. 3:**
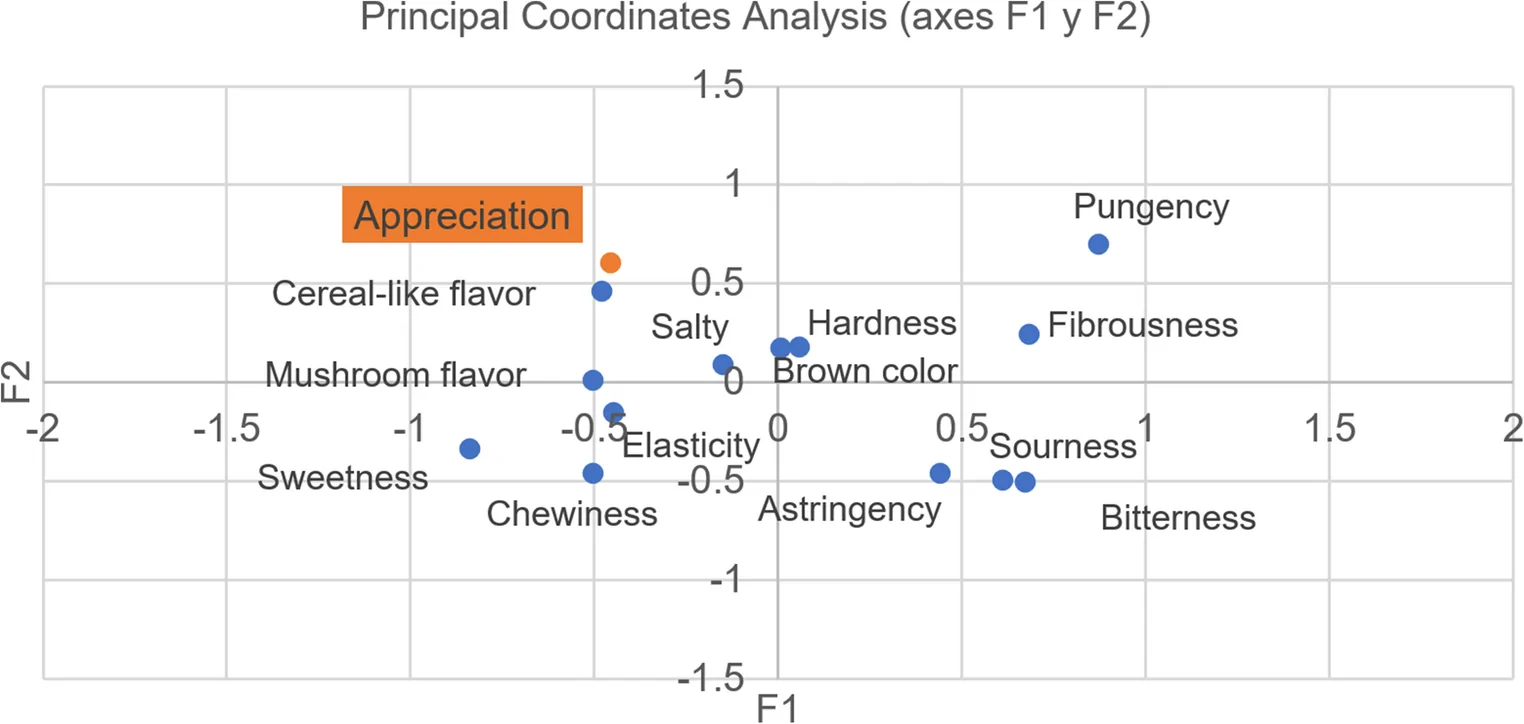
Principal Coordinates Analysis (PCoA) illustrating the association between sensory descriptors and overall appreciation of mushroom samples

Pairwise comparisons using Sheskin’s critical difference procedure showed that *P. ostreatus*, *P. djamor*, and *N. ponderosus* were mainly associated with mushroom-like and cereal-like flavors, as well as chewy textures, attributes positively related to consumer acceptance (Figs. 2 and 3). In contrast, *H. erinaceus* was characterized by bitterness, astringency, and sourness, which negatively affected overall liking. The perceived bitterness may be related to the presence of metabolites, including terpenoids, phenolic compounds, hydrophobic amino acids, and peptides, several of which have been identified as contributors to bitter taste perception in foods [32].

Correlation analysis confirmed that mushroom-like and cereal-like flavors were the main drivers of acceptance, whereas bitterness and astringency were negatively associated with hedonic scores (Fig. 3; Table 3, Supplementary Table 3). Similar relationships have been reported previously for edible mushrooms and mushroom-derived products [18, 33–35].

Correspondence analysis explained 85.12% of the total variability with F1 and F2 accounting for 71.54 and 13.58%, respectively. F1 separated desirable sensory attributes (sweetness, cereal-like flavor, mushroom flavor, chewiness) from undesirable attributes (bitterness, pungency, astringency, sourness, and fibrousness). Accordingly, *P. ostreatus*, *P. djamor*, and *L. edodes* were associated with favorable sensory characteristics, whereas *H. erinaceus* was linked to bitterness and astringency (Fig. 2).

**Fig. 4:**
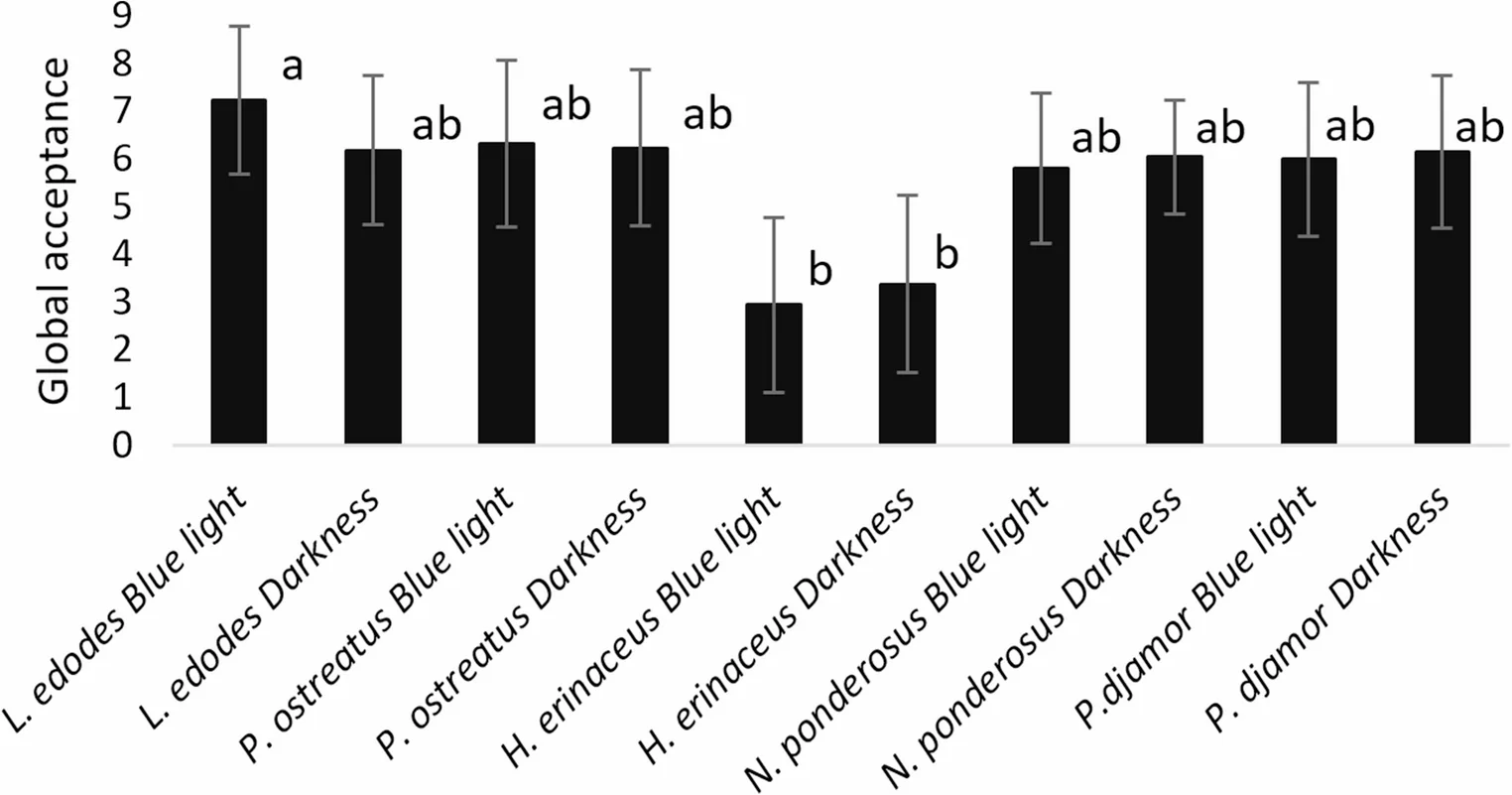
Mean overall hedonic acceptance scores (9-point scale) for each mushroom species under blue-light exposure and darkness. Error bars represent standard deviation among panelists. Different letters indicate significant differences among samples according to one-way ANOVA followed by Tukey’s *post hoc* test, *n* = 21, *p* = 0.05

Sensory differences between light and darkness treatments were less pronounced than the morphological changes observed in fresh mushrooms, suggesting that dehydration and rehydration attenuated some cultivation-induced effects. Nevertheless, blue-light cultivation slightly reduced mushroom-like and cereal-like flavor intensity in *P. ostreatus*, *P. djamor*, and *N. ponderosus*, resulting in a more homogeneous sensory profile and were clearly differentiated from their counterparts cultivated in darkness (Fig. 2). Despite these changes, mushrooms cultivated under blue light maintained high consumer acceptance, particularly *L. edodes*, *P. ostreatus*, and *P. djamor* (Fig. 4). Among all treatments, blue-light-grown *L. edodes* achieved the highest hedonic score (was considered as the “Ideal sample”), whereas *H. erinaceus* showed the lowest acceptance, indicating the need for further optimization of cultivation conditions or postharvest processing strategies to improve its sensory quality.

## Conclusions

Blue-light exposure significantly influenced the productivity, morphology, phenolic content, and sensory properties of edible mushrooms, although responses were species dependent. The greatest productive response was observed in *P. djamor*, while *P. ostreatus* and *H. erinaceus* also showed improved yields. Blue light enhanced total phenolic content in some species but had limited effects on antioxidant activity.

Sensory evaluation indicated that mushroom-like and cereal-like flavors were the main drivers of consumer acceptance, whereas bitterness and astringency negatively affected preference. *Lentinula edodes*, *P. ostreatus*, and *P. djamor* exhibited the highest acceptance and commercial potential.

Overall, blue-light cultivation represents a sustainable strategy to improve mushroom productivity and selected quality attributes without compromising consumer acceptance. Furthermore, the application of CATA proved valuable in linking cultivation conditions to consumer perception, thereby supporting the development of cultivation protocols that enhance both the functional quality and marketability of edible mushrooms.

## Supplementary Information

Below is the link to the electronic supplementary material.

Supplementary Material 1

Supplementary Material 2

## Data Availability

No datasets were generated or analysed during the current study.

## References

[1] Rojas-Martínez R, Aguilar-Salinas CA, Romero-Martínez M et al (2021) Trends in the prevalence of metabolic syndrome and its components in Mexican adults, 2006–2018. Salud Pública Mex 563:713–724. 10.21149/12835 35099910

[2] Grimm D, Wösten H (2018) Mushroom cultivation in the circular economy. Appl Microbiol Biotechnol 102:7795–7803. 10.1007/s00253-018-9226-8 PMC6132538 30027491

[3] Liu J, Sun J, He R, Xia J et al (2024) The situation of counterfeited and mislabeled commercialized edible mushrooms in China and the development of possible controls. Foods 13(19):3097. 10.3390/foods13193097 PMC11476016 39410130

[4] Pérez-Moreno J, Mortimer PE, Xu J et al (2021) Global perspectives on the ecological, cultural, and socioeconomic relevance of wild edible fungi. Stud Fungi 6(1):408–424. 10.5943/sif/6/1/31

[5] Arshadi N, Nouri H, Moghimi H (2023) Increasing the production of the bioactive compounds in medicinal mushrooms: an omics perspective. Microb Cell Fact 22(1):11. 10.1186/s12934-022-02013-x PMC9841694 36647087

[6] González-Tijera M, Mata G, Trigos Á et al (2024) Producción del hongo del maguey *Pleurotus agaves* en sustratos formulados. Sci Fungorum 55:e1466. 10.33885/sf.2024.55.1466

[7] Arana-Gabriel Y, Burrola-Aguilar C, Franco-Maass S et al (2020) Characterization and evaluation of *Flammulina mexicana* growth in lignocellulosic residues. Bosque (Valdivia) 41(2):173–182. 10.4067/S0717-92002020000200173

[8] Xu Y, Huang XM, Wang ZX et al (2025) Blue Light Receptor WC-2 Regulates Ganoderic Acid Biosynthesis in *Ganoderma lingzhi*. J Fungi (Basel) 11(9):646. 10.3390/jof11090646 PMC12470545 41003192

[9] Kumla J, Suwannarach N, Sujarit K et al (2020) Cultivation of Mushrooms and Their Lignocellulolytic Enzyme Production Through the Utilization of Agro-Industrial Waste. Molecules (Basel Switz 25(12):2811. 10.3390/molecules25122811 PMC7355594 32570772

[10] Yu Z, Fischer R (2019) Light sensing and responses in fungi. Nat Rev Microbiol 17:27. 10.1038/s41579-018-0109-x 30377305

[11] Cheng CK, Au CH, Wilke SK et al (2013) 5¨-Serial Analysis of gene expression studies reveal a transcriptomic switch during fruiting body development in *Coprinopsis cinerea*. BMC Genomics 14:195. 10.1186/1471-2164-14-195 PMC3606632 23514374

[12] Wenbing CX, Zhu GZ, Zhenxiu LY et al (2018) Comparative transcriptomics of *Pleurotus eryngi*i reveals blue-light regulation of carbohydrate-active enzymes (CAZymes) expression at primordium differentiated into fruiting body stage. Genomics 110(3):201–209. 10.1016/j.ygeno.2017.09.012 28970048

[13] Zhang J, Ren A, Chen H et al (2015) Transcriptome Analysis and Its Application in Identifying Genes Associated with Fruiting Body Development in Basidiomycete *Hypsizygus marmoreus*. PLoS ONE 10(4):e0123025. 10.1371/journal.pone.0123025 PMC4383556 25837428

[14] Mykchaylova O, Dubova H, Negriyko A et al (2024) Photoregulation of the biosynthetic activity of the edible medicinal mushroom *Lentinula edodes* in vitro. Photochem Photobiol Sci 23:435–449. 10.1007/s43630-023-00529-8 38289457

[15] Huang MY, Lin KH, Lu CC et al (2017) The intensity of blue light-emitting diodes influences the antioxidant properties and sugar content of oyster mushrooms (*Lentinus sajor-caju*). Sci Hort 218:14: 8–13. 10.1016/j.scienta.2017.02.014

[16] Kojima M, Kimura N, Miura R (2015) Regulation of Primary Metabolic Pathways in Oyster Mushroom Mycelia Induced by Blue Light Stimulation: Accumulation of Shikimic Acid. Sci Rep 5:8630. 10.1038/srep08630. PMC4342562 25721093

[17] Mykchaylova O, Lomberg M, Galkin A, Poyedinok N (2025) Regulation of the biological activity of medicinal macromycetes using low- intense quasi-monochromatic and laser light. Wild edible plants: Improving foods nutritional value and human health through biotechnology. CRC, Boca Raton, pp 398–430. 10.1201/9781003486794-14

[18] Omarini A, Nepote V, Grosso NR et al (2010) Sensory analysis and fruiting bodies characterisation of the edible mushrooms *Pleurotus ostreatus* and *Polyporus tenuiculus* obtained on leaf waste from the essential oil production industry. Int J Food Sci Technol 45:466–474. 10.1111/j.1365-2621.2009.02147.x

[19] Yue Z, Zhang W, Liu W et al (2022) Effect of Different Light Qualities and Intensities on the Yield and Quality of Facility-Grown *Pleurotus eryngii*. J Fungi 8:1244. 10.3390/jof8121244 PMC9786600 36547577

[20] Atila F (2019) The Use of Phenolic-rich Agricultural Wastes for *Hericium erinaceus* and *Lentinula edodes* Cultivation, Ege Üniv. Ziraat Fak Derg 56(4):417–425. 10.20289/zfdergi.528957

[21] Zhou Z, Cheng G, Chen et al (2025) Utilizing Agrobyproducts: Potential Alternative Substrates for Cultivation of *Lentinula edodes*. Fermentation 11(5):245. 10.3390/fermentation11050245

[22] International Organization for Standardization (1991) *ISO 3972:1991. Sensory analysis — Methodology — Method of investigating sensitivity of taste*

[23] International Organization for Standardization (1992) *ISO 5496:1992. Sensory analysis — Methodology — Initiation*

[24] Zhu Y, Jia C, Wang C (2024) Yield increment and transcriptome response caused by blue light treatment in Hericium coralloides. BMC Genomics 25:1244. 10.1186/s12864-024-11108-1 PMC11668087 39719598

[25] Im JH, Park CH, Shin JH et al (2024) Effects of Light on the Fruiting Body Color and Differentially Expressed Genes in *Flammulina velutipes*. J Fungi (Basel). 22;10(6):372. 10.3390/jof10060372 PMC11204606 38921359

[26] Whitmore C, Coon D, Rodriguez B et al (2025) Impact of Light Spectra and Substrate Composition on the Bioefficiency, Nutritional Content, and Morphology of Oyster Mushrooms. Horticulturae 11(12):1430. 10.3390/horticulturae11121430

[27] Lu Z, Yang Y, Hu S et al (2025) The Agronomic Traits Differences in *Hericium erinaceus* Cultivated with Different Straw Formulations by Replacing Wood with Straw. Horticulturae 11(10):1220. 10.3390/horticulturae11101220

[28] Wang H, Tong X, Tian F et al (2020) Transcriptomic profiling sheds light on the blue-light and red-light response of oyster mushroom (Pleurotus ostreatus). AMB Express 10:10. 10.1186/s13568-020-0951-x PMC6969877 31955301

[29] Zhu L, Su Y, Ma S et al (2024) Comparative proteomic analysis reveals candidate pathways related to the effect of different light qualities on the development of mycelium and fruiting body of *Pleurotus ostreatus*. J Agric Food Chem 72:1361–1375. 10.1021/acs.jafc.3c06083 38166381

[30] Tang J, Shen H, Lv W et al (2025) Edible Fungi Melanin: Recent Advances in Extraction, Characterization, Biological Activity and Applications. J Fungi 11:738. 10.3390/jof11100738 PMC12565410 41149928

[31] Sánchez C (2016) Reactive oxygen species and antioxidant properties from mushrooms. Synth Syst Biotechnol 2(1):13–22. 10.1016/j.synbio.2016.12.001 PMC5625788 29062957

[32] Qiao K, Zhao M, Huang Y et al (2024) Bitter perception and effects of foods rich in bitter compounds on human health: a comprehensive review. Foods 13(23):3747. 10.3390/foods13233747 PMC11640738 39682819

[33] Chun S, Chambers E, Han I (2020) Development of a sensory flavor lexicon for mushrooms and subsequent characterization of fresh and dried mushrooms. *Foods* 9(8):980. 10.3390/foods9080980 PMC7466268 32718026

[34] Van-Dam L, Cruz-Morales P, Rodriguez VN et al (2024) GastronOmics: Edibility and safety of mycelium of the oyster mushroom *Pleurotus ostreatus*. Curr Res Food Sci 9:100866. 10.1016/j.crfs.2024.100866 PMC11490876 39429921

[35] Srivastava A, Attri BL, Bijla S et al (2025) Utilization of lion’s mane mushroom (*Hericium erinaceus*) for vegan crab analogue: effects of pre-treatments on nutritional, sensory, and physicochemical properties. J Food Sci Technol. 10.1007/s13197-025-06483-w

