# Supplementary material for: Blue-Light Exposure Enhances Productivity, Total Phenolic Content, and Sensory Attributes in Edible Mushrooms: A Multispecies Study
Source: Plant Foods Hum Nutr. 2026 Aug 6;81(3):104. doi: 10.1007/s11130-026-01555-4 (PMC13447471; doi:10.1007/s11130-026-01555-4)
Supplement: Supplementary file 1 [file 11130_2026_1555_MOESM1_ESM.docx]

**SUPPLEMENTARY MATERIAL**

**Blue-Light Exposure Enhances Productivity, Total Phenolic Content, and Sensory Attributes in Edible Mushrooms: A Multispecies Study**

Atahualpa Guzmán-de-Casa^1^. Eréndira Valencia-Avilés^2^. Pedro A. García-Saucedo^3^. Erick Sierra-Campos^4^*.* Teresita del Carmen Ávila-Val^3^. Víctor Gómez-Reyes^5^. Víctor Olalde-Portugal^1^

^1^Centro de Investigaciones Avanzadas, Irapuato. Irapuato Guanajuato 36824, México

^2^Universidad Politécnica de Uruapan, Uruapan, Michoacán 60210, México

^3^Facultad de Agrobiología ‘Presidente Juárez’. Universidad Michoacana de San Nicolás de Hidalgo. Uruapan, Michoacán 60040. México

^4^Facultad de Ciencias Químicas. Universidad Juárez de Durango, Gómez Palacio, Durango 35010. Mexico

^5^Facultad de Biología. Universidad Michoacana de San Nicolás de Hidalgo. Morelia, Michoacán 58030. Mexico

Corresponding authors: Víctor Olalde Portugal, Atahualpa Guzmán de Casa

[victor.olalde@cinvestav.mx](mailto:victor.olalde@cinvestav.mx) ; [atahualpa.guzman@umich.mx](mailto:atahualpa.guzman@umich.mx)

**1. Materials and methods**

**1.1. Substrates, Inoculation, and Cultivation Conditions**

Cultivation was carried out on lignocellulosic substrates commonly used for edible mushroom production. *Hericium erinaceus*, *Pleurotus ostreatus*, *Pleurotus djamor*, and *Lentinula edodes* were cultivated on composted oak (*Quercus* spp.) sawdust, a substrate widely employed for the production of ligninolytic mushrooms due to its favorable physicochemical characteristics and its suitability for fungal degradation of cellulose, hemicellulose, and lignin (1). To enhance nutrient availability, oak sawdust was composted for two weeks and supplemented with wheat bran (15%, w/w), resulting in an estimated carbon-to-nitrogen ratio of 50–60:1.

Because *N. ponderosus* naturally colonizes pine wood in temperate forests, this species was cultivated on a 1:1 (w/w) mixture of composted oak and pine sawdust supplemented with 15% (w/w) wheat bran. The moisture content of all substrates was adjusted to 60% before sterilization.

Substrate portions (1.5 kg fresh weight) were packed into polypropylene bags, sterilized at 121 °C for 90 min, cooled to room temperature, and inoculated with 5 g of sorghum grain spawn. The inoculated bags were incubated in darkness until the substrate was fully colonized by the mycelium.

Following mycelial colonization, fruiting was induced either in continuous darkness or under blue fluorescent light (25 μmol m⁻² s⁻¹, 8 h photoperiod) at 80–90% relative humidity. Artificial illumination is routinely applied during commercial mushroom cultivation to stimulate primordium formation and fruiting body development, particularly in species of *Hericium* and *Lentinula* (1, 2). Therefore, blue light and darkness were evaluated as cultivation conditions to determine their effects on mushroom productivity, total phenolic content, antioxidant activity, and sensory characteristics. Fruiting bodies were harvested at commercial maturity and dehydrated at 45 °C for 16 h before analysis.

**1.2 Sensory Evaluation and CATA Analysis**

Omarini et al (3) used trained descriptive sensory analysis and found that *P. ostreatus* grown on aromatic plant residues showed changes in both yield and sensory properties. Sensory evaluation methods, such as descriptive sensory analysis, require extensive panel training (4). Alternatively, the Check-All-That-Apply (CATA) methodology offers a rapid and cost-effective approach using semi-trained or untrained panelists to detect sensory differences (5). Panel selection can be optimized using 6-n-propylthiouracil (PROP) sensitivity tests, which correlate with the perception of basic tastes and certain oral sensations (6-7).

**1.2.1 Semi-trained Panel Recruitment and Training**

Sensory evaluation was conducted with a semi-trained panel. Participants were screened for bitterness sensitivity using 6-n-propylthiouracil (PROP), and only those with “high” or “very high” perception were included. Training followed ISO 3972:1991 (8) and ISO 5496:1992 (9) guidelines for taste recognition and sensory discrimination, including the use of intensity scales and product-specific descriptors.

**1.2.2 Sample Preparation for Sensory Testing**

For sensory evaluation, dehydrated samples were rehydrated in 0.75% (w/v) saline solution at 80 °C for 40 min (salt:mushroom:water ratio 0.75:10:100), drained, coded, and presented in randomized order (10). Sensory descriptors were adapted from Omarini et al. (3) and included appearance (brown color), flavor (cereal-like and mushroom flavors), mouthfeel (astringency and pungency), basic tastes (sweetness, saltiness, bitterness, and sourness), and texture (hardness, fibrousness, elasticity, and chewiness). Such attributes were evaluated using the Check-All-That-Apply (CATA) method, followed by intensity scoring and overall acceptance on a 9-point hedonic scale.

**2. Additional results**

**2.1 Sensory Analysis and CATA**

**2.1.1 Panel Composition**

A semi-trained panel was assembled from students and academic staff across three institutions. From 127 initial participants, screening through a sociodemographic survey, PROP bitterness sensitivity test, basic taste identification tests, and linear scale training resulted in the selection of 36 judges (26 women, 10 men). Following hedonic screening, 9 individuals were excluded due to expressed dislike of mushrooms. The final CATA evaluation was conducted by 27 semi-trained panelists (17 women, 10 men; age range: 17–44 years).

**2.1.2 Frequency of Perceived Attributes**

Across the 13 evaluated attributes, panelists selected between 27% and 60% of attributes per sample. The least frequently selected attributes were pungency (7%), sweetness (18%), and sourness (19%), while the most frequently selected were brown color (79%) and mushroom flavor (54%). Other frequently selected attributes included salty taste (54%), astringency (47%), elastic texture (47%), chewiness (46%), and cereal-like flavor (41%) (Table 1).

**Table 1.** Frequency (%) of CATA attribute selection for rehydrated mushroom samples cultivated under blue-light exposure and in darkness^a^

| Attribute | Count | % |
| --- | --- | --- |
| Sweetness | 49 | 18% |
| Salty | 145 | 54% |
| Bitterness | 105 | 39% |
| Sourness | 52 | 19% |
| Cereal-like flavor | 110 | 41% |
| Mushroom flavor | 147 | 54% |
| Brown color | 212 | 79% |
| Astringency | 126 | 47% |
| Pungency | 19 | 7% |
| Hardness | 103 | 38% |
| Fibrousness | 107 | 40% |
| Elasticity | 127 | 47% |
| Chewiness | 124 | 46% |

^a^Counts represent the total number of selections across all panelists and samples; percentages represent the proportion of panelists selecting each attribute

Cochran's Q test identified bitterness, sourness, cereal-like flavor, mushroom flavor, brown color, astringency, fibrousness, and chewiness as the sensory attributes that significantly discriminated among the samples (p < 0.05), whereas sweetness, pungency, hardness, and elasticity showed no significant differences (Table 2).

**Table 2** Cochran’s Q test results for CATA attributes across mushroom samples^a^

| **Attribute** | **p-value** |
| --- | --- |
| Sweetness | 0.030 |
| Salty | 0.003 |
| Bitterness | <0.0001 |
| Sourness | 0.001 |
| Cereal-like flavor | <0.0001 |
| Mushroom flavor | <0.0001 |
| Brown color | <0.0001 |
| Astringency | 0.000 |
| Pungency | 0.077 |
| Hardness | 0.022 |
| Fibrousness | <0.0001 |
| Elasticity | 0.221 |
| Chewiness | <0.0001 |

^a^Attributes with *p* < 0.05 significantly discriminated among samples

**2.1.3 Correlation among sensory attributes and appreciation**

**Table 3** Correlation matrix of sensory descriptors and overall appreciation for the evaluated mushroom species^a^

|  | Sweetness | Salty | Bitterness | Sourness | Cereal-like flavor | Mushroom | Brown color | Astringency | Pungency | Hardness | Fibrousness | Elasticity | Chewiness | Appreciation |
| --- | --- | --- | --- | --- | --- | --- | --- | --- | --- | --- | --- | --- | --- | --- |
| Sweetness | **1** | 0.197 | -0.216 | -0.245 | 0.292 | 0.309 | 0.059 | -0.377 | -0.873 | 0.176 | -0.195 | 0.455 | 0.250 | 0.187 |
| Salty | 0.197 | **1** | 0.006 | -0.221 | -0.035 | -0.049 | -0.104 | -0.001 | 0.086 | 0.195 | -0.192 | 0.078 | 0.248 | 0.136 |
| Bitterness | -0.216 | 0.006 | **1** | 0.330 | -0.357 | -0.317 | -0.154 | 0.506 | 0.300 | -0.036 | 0.360 | -0.136 | -0.104 | -0.456 |
| Sourness | -0.245 | -0.221 | 0.330 | **1** | -0.539 | -0.299 | 0.015 | 0.313 | 0.149 | 0.076 | 0.238 | 0.042 | -0.156 | -0.288 |
| Cereal-like flavor | 0.292 | -0.035 | -0.357 | -0.539 | **1** | -0.098 | 0.187 | -0.064 | -0.260 | -0.074 | -0.125 | 0.195 | 0.018 | 0.190 |
| Mushroom | 0.309 | -0.049 | -0.317 | -0.299 | -0.098 | **1** | -0.170 | -0.143 | -0.306 | -0.056 | -0.229 | 0.170 | 0.262 | 0.291 |
| Brown color | 0.059 | -0.104 | -0.154 | 0.015 | 0.187 | -0.170 | **1** | 0.009 | -0.052 | 0.074 | -0.022 | -0.006 | -0.101 | -0.063 |
| Astringency | -0.377 | -0.001 | 0.506 | 0.313 | -0.064 | -0.143 | 0.009 | **1** | -0.149 | -0.057 | 0.200 | -0.146 | -0.006 | -0.275 |
| Pungency | -0.873 | 0.086 | 0.300 | 0.149 | -0.260 | -0.306 | -0.052 | -0.149 | **1** | 0.171 | 0.520 | -0.220 | -0.481 | -0.078 |
| Hardness | 0.176 | 0.195 | -0.036 | 0.076 | -0.074 | -0.056 | 0.074 | -0.057 | 0.171 | **1** | -0.150 | -0.116 | -0.138 | 0.005 |
| Fibrousness | -0.195 | -0.192 | 0.360 | 0.238 | -0.125 | -0.229 | -0.022 | 0.200 | 0.520 | -0.150 | **1** | -0.497 | -0.445 | -0.122 |
| Elasticity | 0.455 | 0.078 | -0.136 | 0.042 | 0.195 | 0.170 | -0.006 | -0.146 | -0.220 | -0.116 | -0.497 | **1** | 0.018 | 0.136 |
| Chewiness | 0.250 | 0.248 | -0.104 | -0.156 | 0.018 | 0.262 | -0.101 | -0.006 | -0.481 | -0.138 | -0.445 | 0.018 | **1** | 0.095 |
| Appreciation | 0.187 | 0.136 | -0.456 | -0.288 | 0.190 | 0.291 | -0.063 | -0.275 | -0.078 | 0.005 | -0.122 | 0.136 | 0.095 | **1** |

^a^The correlation matrix was used as the input dataset for principal component analysis (PCA)

**2.1.4 Sensory profile**

*L. edodes* cultivated under blue light exhibited greater elasticity, chewiness, astringency, and a more pronounced brown coloration compared to samples grown in darkness. In *P. djamor*, exposure to blue light resulted in increased hardness and reduced elasticity and chewiness. In contrast, no significant differences were observed between blue light and dark incubation conditions in *P. ostreatus*, *N. ponderosus*, and *H. erinaceus*.

|  | L.edodes Blue Light | L. edodes Darkness | P.ostretus Blue Light | P.ostreatus Darness | P.djamor Blue Light | P. djamor Darkness | H. erinaceus Blue Light | H. erinaceus Darkness | N. ponderosus Blue Light | N. ponderosus Darkness |
| --- | --- | --- | --- | --- | --- | --- | --- | --- | --- | --- |
| Sweetness | 0.169231 | 0.415385 | 0.488462 | 0.742308 | 0.607692 | 0.496154 | 0.0346154 | 0.196154 | 0.688462 | 0.596154 |
| Salty | 4.03077 | 4.60769 | 4.24615 | 2.24231 | 5.06154 | 4.52692 | 1.51154 | 2.11538 | 2.13077 | 0.942308 |
| Bitterness | 1.48846 | 2.68846 | 1.46923 | 0.346154 | 1.16538 | 1.24615 | 10.6269 | 10.45 | 1.33462 | 0.884615 |
| Sourness | 0.930769 | 0.480769 | 0.857692 | 0.638462 | 1.73462 | 0.392308 | 4.84615 | 4.33462 | 0.638462 | 0.442308 |
| Cereal-like flavor | 2.44615 | 2.24615 | 4.85385 | 4.91923 | 2.45385 | 3.15 | 0.703846 | 1.56923 | 3.07692 | 4.20769 |
| Mushroom flavor | 6.31923 | 5.51154 | 4.83077 | 5.17692 | 4.82692 | 5.10769 | 2.40769 | 1.81923 | 3.78077 | 3.81154 |
| Brown color | 4.86923 | 1.72308 | 7.45385 | 7.76923 | 4.58846 | 3.78462 | 5.01154 | 5.91923 | 9.83462 | 8.95769 |
| Astringency | 4.13846 | 2.2 | 2.43462 | 1.61154 | 1.56538 | 1.86923 | 6.87308 | 6.75 | 2.60385 | 1.60385 |
| Pungency | 0.115385 | 0.0307692 | 0.538462 | 0 | 0.1 | 0.334615 | 1.18846 | 0.765385 | 0.142308 | 0.334615 |
| Hardness | 0.9 | 2.73077 | 2.20385 | 2.98077 | 5.10385 | 2.86538 | 2.23462 | 1.69615 | 2.79231 | 5.73846 |
| Fibrousness | 0.634615 | 2.91923 | 2.39231 | 3.33846 | 2.78846 | 1.5 | 7.61154 | 7.20385 | 1.77692 | 3.93846 |
| Elasticity | 5.18077 | 2.71923 | 3.71154 | 3.45385 | 2.70769 | 4.81923 | 2.20385 | 3.43846 | 3.73077 | 3.22692 |
| Chewiness | 5.78846 | 3.73462 | 4.26923 | 3.73462 | 3.96923 | 4.94231 | 1.25769 | 0.615385 | 3.3 | 2.12308 |

|  | L.edodes Blue Light | L. edodes Darkness |
| --- | --- | --- |
| Sweetness | 0.169231 | 0.415385 |
| Salty | 4.03077 | 4.60769 |
| Bitterness | 1.48846 | 2.68846 |
| Sourness | 0.930769 | 0.480769 |
| Cereal-like flavor | 2.44615 | 2.24615 |
| Mushroom flavor | 6.31923 | 5.51154 |
| Brown color | 4.86923 | 1.72308 |
| Astringency | 4.13846 | 2.2 |
| Pungency | 0.115385 | 0.0307692 |
| Hardness | 0.9 | 2.73077 |
| Fibrousness | 0.634615 | 2.91923 |
| Elasticity | 5.18077 | 2.71923 |
| Chewiness | 5.78846 | 3.73462 |

|  | P.ostretus Blue Light | P.ostreatus Darness |
| --- | --- | --- |
| Sweetness | 0.488462 | 0.742308 |
| Salty | 4.24615 | 2.24231 |
| Bitterness | 1.46923 | 0.346154 |
| Sourness | 0.857692 | 0.638462 |
| Cereal-like flavor | 4.85385 | 4.91923 |
| Mushroom flavor | 4.83077 | 5.17692 |
| Brown color | 7.45385 | 7.76923 |
| Astringency | 2.43462 | 1.61154 |
| Pungency | 0.538462 | 0 |
| Hardness | 2.20385 | 2.98077 |
| Fibrousness | 2.39231 | 3.33846 |
| Elasticity | 3.71154 | 3.45385 |
| Chewiness | 4.26923 | 3.73462 |

|  | H. erinaceus Blue Light | H. erinaceus Darkness |
| --- | --- | --- |
| Sweetness | 0.0346154 | 0.196154 |
| Salty | 1.51154 | 2.11538 |
| Bitterness | 10.6269 | 10.45 |
| Sourness | 4.84615 | 4.33462 |
| Cereal-like flavor | 0.703846 | 1.56923 |
| Mushroom flavor | 2.40769 | 1.81923 |
| Brown color | 5.01154 | 5.91923 |
| Astringency | 6.87308 | 6.75 |
| Pungency | 1.18846 | 0.765385 |
| Hardness | 2.23462 | 1.69615 |
| Fibrousness | 7.61154 | 7.20385 |
| Elasticity | 2.20385 | 3.43846 |
| Chewiness | 1.25769 | 0.615385 |

|  | P.djamor Blue Light | P. djamor Darkness |
| --- | --- | --- |
| Sweetness | 0.607692 | 0.496154 |
| Salty | 5.06154 | 4.52692 |
| Bitterness | 1.16538 | 1.24615 |
| Sourness | 1.73462 | 0.392308 |
| Cereal-like flavor | 2.45385 | 3.15 |
| Mushroom flavor | 4.82692 | 5.10769 |
| Brown color | 4.58846 | 3.78462 |
| Astringency | 1.56538 | 1.86923 |
| Pungency | 0.1 | 0.334615 |
| Hardness | 5.10385 | 2.86538 |
| Fibrousness | 2.78846 | 1.5 |
| Elasticity | 2.70769 | 4.81923 |
| Chewiness | 3.96923 | 4.94231 |

|  | N. ponderosus Blue Light | N. ponderosus Darkness |
| --- | --- | --- |
| Sweetness | 0.688462 | 0.596154 |
| Salty | 2.13077 | 0.942308 |
| Bitterness | 1.33462 | 0.884615 |
| Sourness | 0.638462 | 0.442308 |
| Cereal-like flavor | 3.07692 | 4.20769 |
| Mushroom flavor | 3.78077 | 3.81154 |
| Brown color | 9.83462 | 8.95769 |
| Astringency | 2.60385 | 1.60385 |
| Pungency | 0.142308 | 0.334615 |
| Hardness | 2.79231 | 5.73846 |
| Fibrousness | 1.77692 | 3.93846 |
| Elasticity | 3.73077 | 3.22692 |
| Chewiness | 3.3 | 2.12308 |

**Fig. 1** Sensory profile of the evaluated samples measured using a structured 15 cm intensity scale. The blue line represents mushrooms cultivated under blue light exposure, whereas the black line represents mushrooms cultivated in darkness

**3. References**

1. Zhou, Z., Cheng, G., Chen, W et al (2025) Utilizing Agrobyproducts: Potential Alternative Substrates for Cultivation of *Lentinula edodes*. *Fermentation*, *11*(5), 245. <https://doi.org/10.3390/fermentation11050245>
2. Atila, F (2019) The use of phenolic-rich agricultural wastes for Hericium erinaceus and Lentinula edodes cultivation and its effect on yield performance. *Journal of Agriculture Faculty of Ege University*, *56*(4), 417-425. [https://doi.org/10.20289/zfdergi.528957](https://doi.org/10.20289/zfdergi.528957)
3. Omarini A, Nepote V, Grosso NR et al (2010) “Sensory analysis and fruiting bodies characterisation of the edible mushrooms *Pleurotus ostreatus* and *Polyporus tenuiculus* obtained on leaf waste from the essential oil production industry”. International Journal of Food Science and Technology,45, 466–474 doi:10.1111/j.1365-2621.2009.02147.x
4. Hugi A, Voirol E (2001) Instrumental Measurements and Sensory Parameters. *Instrumentation and Sensors for the Food Industry* 31–60. DOI:[10.1533/9781855736481.31](https://doi.org/10.1533/9781855736481.31)
5. Meyners, M., John C. C, Thomas C (2013) “Existing and New Approaches for the Analysis of CATA Data.” *Food Quality and Preference* 30(2):309–19. DOI:[10.1016/j.foodqual.2013.06.010](https://doi.org/10.1016/j.foodqual.2013.06.010)
6. Bartoshuk LM, Duffy VB, Lucchina LA et al (1998) PROP (6-n-Propylthiouracil) Supertasters and the Saltiness of NaCl. *Annals of the New York Academy of Sciences* 855:793–96. <https://doi.org/10.1111/j.1749-6632.1998.tb10660.x>
7. Chang WI, Chung JW, Kim YK, Chung SC, Kho HS (2006) The relationship between phenylthiocarbamide (PTC) and 6-n-propylthiouracil (PROP) taster status and taste thresholds for sucrose and quinine. Arch Oral Biol. May;51(5):427-32. doi: 10.1016/j.archoralbio.2005.10.002
8. International Organization for Standardization (1991) *ISO 3972:1991. Sensory analysis — Methodology — Method of investigating sensitivity of taste*
9. International Organization for Standardization (1992) *ISO 5496:1992. Sensory analysis — Methodology — Initiation and training of assessors in the detection and recognition of odours*
10. Boin ASFE, Azevedo MAM C., Nunes MSA J et al (2016) Consumer Acceptability and Descriptive Characterization of Fresh and Dried King Oyster (*Pleurotus eryngii*) and Hedgehog (*Hydnum repandum*) Mushrooms. Journal of Food Research; Vol. 5, No. 4; ISSN 1927-0887. http://dx.doi.org/10.5539/jfr.v5n4p55
